# Contemporary Analysis of Inconsistencies Between Physician-reported Disclosures at the AAOS Annual Meeting and Industry-reported Financial Disclosures in the Open Payments Database

**DOI:** 10.5435/JAAOSGlobal-D-22-00048

**Published:** 2022-07-06

**Authors:** Patawut Bovonratwet, Wasif Islam, Evan L. Honig, Brooks M. Martino, Aaron Z. Chen, Todd J. Albert, Edwin P. Su

**Affiliations:** From the Adult Reconstruction and Joint Replacement Service, Hospital for Special Surgery (Dr. Bovonratwet, Dr. Su); the Weill Cornell Medical College (Islam, Honig, Martino, and Chen); and the Department of Spine Surgery, Hospital for Special Surgery, New York, NY (Dr. Albert).

## Abstract

**Introduction:**

Healthcare regulators and patients are increasingly interested in financial transparency between physicians and the industry because of concerns of bias.

**Methods:**

Disclosures for every first and last author with a medical degree from the United States associated with a poster or podium presentation at the American Academy of Orthopaedic Surgeons (AAOS) 2019 Annual Meeting were identified. Author characteristics were collected. AAOS disclosures were then compared with disclosures from the Open Payments Database to determine whether any inconsistencies existed.

**Results:**

In total, 2,503 AAOS presenters were identified, and 1,380 authors met the inclusion criteria. Using AAOS disclosures as the standard comparator, 482 authors (35%) had an inconsistency in any category between AAOS disclosures and the Open Payments Database. Inconsistency rates for each category were 8% for royalties, 10% for speaker's fee, 15% for paid consultant, 16% for research, 14% for stocks, and 1% for other financial support.

**Discussion:**

Although the inconsistency rate for each category has improved over the years, the overall inconsistency rate between physician-reported disclosures at a recent AAOS Annual Meeting and industry-reported relationships reported in the Open Payments Database was still 35%.

Driven by a mutual desire to advance surgical care and clinical outcomes of patients, orthopaedic surgery has maintained a long-standing relationship with the industry.^1,2^ This relationship has led to numerous scientific advances that have greatly affected patient care, including the development of technologies, such as implantable devices, imaging modalities, and biologics.^1,2^ Because of these successes, the orthopaedic implant industry has evolved into a 10-billion dollar market in the United States and a 25-billion dollar market worldwide.^3^ Recent studies have demonstrated that orthopaedic surgery receives the most industry payments of any medical specialty.^4,5^

To increase transparency between physicians and the industry, the Physician Payments Sunshine Act, which makes industry payments publicly available in the online Open Payments Database, was enacted in 2010.^6^ After August 2013, this law required industry companies, such as pharmaceutical, medical device, and biologic suppliers, to disclose to the Centers for Medicare and Medicaid Services payments greater than 10 dollars to physicians and academic hospitals.^6^ Specific to orthopaedic surgery, the Open Payments Database contains more than 12,000 orthopaedic surgeons and nearly 60,000 industry payments.^7^ It is worth noting that payments to physicians on the Open Payments Database are industry-reported and not physician-reported.

In alternative attempts to facilitate transparency, the American Academy of Orthopaedic Surgeons (AAOS) has required orthopaedic surgeons presenting at the AAOS Annual Meeting to submit annual conflict of interest disclosures since 1985.^7^ These disclosures detail on an existing relationship between an individual surgeon and an industry supplier, but unlike the Open Payments Database, these do not reveal the monetary amount of the relationship. Furthermore, these disclosures are physician-reported, unlike those in the Open Payments Database.

The AAOS Annual Meetings are the largest orthopaedic gatherings in the United States, and the research presented is often widely disseminated to practicing orthopaedic surgeons, who often find that disclosures influence their interpretation of results.^8^ Therefore, there exists a unique need in reconciling the industry-reported disclosures in the Open Payments Database with the physician-reported disclosures provided in the AAOS Annual Meeting. Thus, the goal of this study was to determine the rates and types of inconsistencies between self-reported disclosures by orthopaedic surgeons at the recent AAOS 2019 Annual Meeting and industry-reported disclosures in the Open Payments Database during the same period.

## Methods

### AAOS 2019 Annual Meeting Data

The AAOS 2019 Annual Meeting was held from March 12 to March 16, 2019, in Las Vegas, Nevada. All attendees at the Annual Meeting who were authors of paper or poster presentations were mandated to disclose “any relevant potentially conflicting interests or commercial relationships.”^9^ The time frame to disclose was “within the 12 months prior to the educational activity.”^10^ Categories for disclosure included "Royalties from a company or supplier,” “Speaker's bureau/paid presentations for a company or supplier,” "Paid employee for a company or supplier,” “Paid consultant for a company or supplier,” “Unpaid consultant for a company or supplier,” “Stock or stock options in a company or supplier,” “Research support from a company or supplier as a PI,” “Other financial or material support from a company or supplier,” “Royalties and financial or material support from publishers,” “Medical/orthopaedic publications' editorial/governing board,” and “Board member/committee appointments for a society” (Table I). Authors with no financial disclosures were required to state that they had no financial disclosures.

**Table 1:** Corresponding AAOS and Open Payments Disclosures Categories

AAOS Disclosure Category	Open Payments Database Disclosure Category
Royalties	Royalty or license
Speaker's bureau or paid presentations	1. Compensation for services other than consulting, including serving as faculty or as a speaker at a venue other than a continuing education program
	2. Compensation for serving as faculty or as a speaker for a nonaccredited and noncertified continuing education program
	3. Compensation for serving as faculty or as a speaker for an accredited or certified continuing education program
	4. Honoraria
Paid consultant	Consulting fees
Research support	1. Research payments
	2. Associated research funding
Stock or stock options	1. Ownership and investment interest
	2. Current or prospective ownership or investment interest
Other financial or material support	1. Charitable contribution
	2. Education
	3. Entertainment
	4. Food and beverage
	5. Gift
	6. Travel or lodging
	7. Grant

AAOS = American Academy of Orthopaedic Surgeons

**Figure 1:**
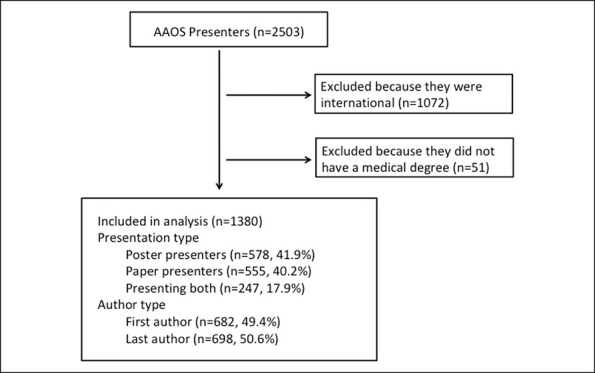
Diagram demonstrating inclusion and exclusion criteria of this study.

Demographic information and disclosures for every first author and last author of a poster or podium presentation at the 2019 Annual Meeting were collected. Authors were classified as poster presenter, podium presenter, or both. Demographic data collected for each author included state of practice, specialty, and whether the author was the first or last author. State of practice was used to categorize authors into regions, which included Northeast, Midwest, South, and West. Self-reported disclosure for each author was extracted from the AAOS disclosure program according to the aforementioned 11 categories.^11^ The number of disclosures within each category and the name of the company for each disclosure were delineated. If the same company was disclosed in different categories by the same author, each occurrence was counted as a separate disclosure. The total number of disclosures for each author was then determined. Initially, 2,503 AAOS presenters were identified. Of these, 1,072 presenters were excluded because of international status and 51 were excluded because of no Doctor of Medicine (M.D.) degree. Only authors with a M.D. degree who practiced in the United States were included because only these individuals had data in the Open Payments Database for comparison. The final cohort consisted of 1,380 US M.D. authors (Figure 1).

### Open Payments Data

The Open Payments Database, which was a part of the Patient Protection and Affordable Care Act (Public Law No. 111-148), contains data collected by the Centers for Medicare and Medicaid Services regarding financial relationships between physicians and manufacturers of drugs, biologics, devices, or medical supplies. Applicable manufacturers are defined as those operating in the United States that manufacture at least one product that is reimbursed by Medicare, Medicaid, or the Children's Health Insurance Program.^12^ All payments or transfers of value greater than $10 to “covered recipients,” who include physicians, dentists, podiatrists, optometrists, or chiropractors, in a given calendar year, must be reported.^7^ Financial data in the Open Payments Database are accessible to the public.^13^

Authors who met the inclusion and exclusion criteria were identified by name in the Open Payments Database. In situations where several physicians have the same name, the author who presented at the AAOS 2019 Annual Meeting was further identified based on his or her specialty as orthopaedic surgery and his or her state of practice in the Open Payments Database.

Data that were extracted from the Open Payments Database included the total value (in dollar amounts) of general payments, of research payments, and of ownership and investment interest from a corresponding time period. For each subgroup of general payments, the name of each unique company reporting a payment in that subgroup was recorded, and the total number of companies that reported a payment for that subgroup was determined. The same methodology was repeated for research payments as well as ownership and investment interest.

### Statistical Comparison of Disclosures

AAOS disclosures for each author included in this study were compared with disclosures that were present in the Open Payments Database. The following AAOS disclosure categories were used for comparison: “Royalties from a company or supplier,” “Speaker's bureau/paid presentations for a company or supplier,” “Paid consultant for a company or supplier,” “Research support from a company or supplier as a PI,” “Stock or stock options in a company or supplier,” and “Other financial or material support from a company or supplier.” Only these AAOS categories were used in the analysis because they directly correspond to a disclosure category in the Open Payments Database (Table 1). The following AAOS disclosures were excluded from the analysis because they did not correspond to any disclosure category in the Open Payments Database: “Paid employee for a company or supplier,” “Unpaid consultant for a company or supplier,” “Royalties and financial or material support from publishers,” “Medical/Orthopaedic publications' editorial/governing board,” and “Board member/committee appointments for a society.”

For the purposes of comparison, the AAOS disclosures were used as the benchmark for which the Open Payments Database was compared against, following the previous methodology.^7^ In other words, for authors with AAOS disclosures, each AAOS disclosure category defined previously was compared with the corresponding disclosure category reported in the Open Payments Database (Table 1 and Table 2) to determine whether any inconsistencies were present. An inconsistency was defined as when a disclosure was present in the AAOS disclosure program but that disclosure was not present in the corresponding category in the Open Payments Database. The overall inconsistency rate was then defined as having at least one inconsistency in any of the previously defined categories of comparison.

**Table 2:** Types of Inconsistencies Between the Open Payments Database and the AAOS Disclosure Database

Type	Inconsistency rate, %
Royalties	7.6
Speaker's bureau or paid presentations	9.6
Paid consultant	15.3
Research support	15.6
Stock or stock options	13.9
Other financial or material support	0.8

AAOS = American Academy of Orthopaedic Surgeons

The chi square or Fisher exact test was used as appropriate to compare the rate of AAOS disclosures and the overall inconsistency rates between regions, specialties, presentation types, author position, and presence of an AAOS disclosure. Next, owing to non-normality of the data determined by the Shapiro-Wilk W test, Mann-Whitney *U* tests were used to compare the number of poster presentations, number of podium presentations, total number of presentations, total number of AAOS disclosures, and total value of payments in the Open Payments Database between authors with versus without inconsistencies. These are univariate analyses used in this study.

Next, independent associations between author characteristics and the presence of an AAOS disclosure were determined using a multivariable Poisson regression with robust error variance. The final multivariable model was selected using a backward stepwise approach, where all variables in Table 1 (excluding presence of an AAOS disclosure due to collinearity) and the number of podium/poster presentations were initially included in the model and variables with the highest *P* values were eliminated one by one until only variables with *P* < 0.05 remained in the model.^14,15^ Variables remaining in the model represented independent author characteristics associated with the presence of an AAOS disclosure. Finally, independent associations between author characteristics and the presence of an inconsistency were determined through the same type of multivariable regression. In this stepwise regression, variables in Table 1, number of podium/poster presentations, number of AAOS disclosures, number of payments in Open Payments Database, and total value of payments in the Open Payments Database are included. These are multivariable analyses used in this study.

### Statistical Comparisons of Total Payments by Specialty

The average total value of payments in the Open Payments Database by specialty was also evaluated. These averages were then compared between specialties through an analysis of variance test.

All statistical analyses were conducted in Stata version 13.1 (StataCorp, LP). The level of significance was set at a 2-sided level of 0.05 (*P* < 0.05).

## Results

### Author Characteristics

**Figure 2:**
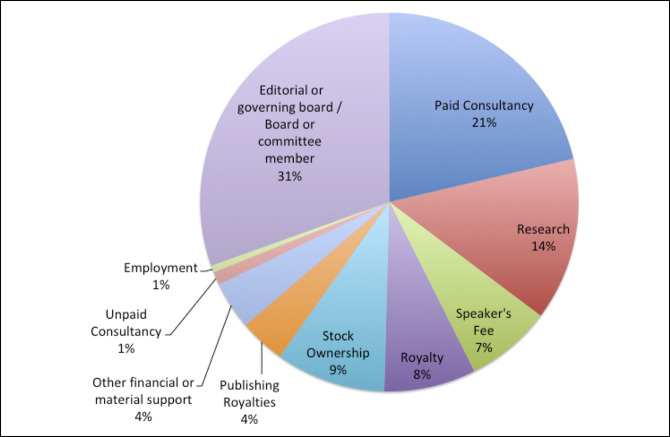
Illustration showing the distribution of AAOS disclosures.

**Figure 3:**
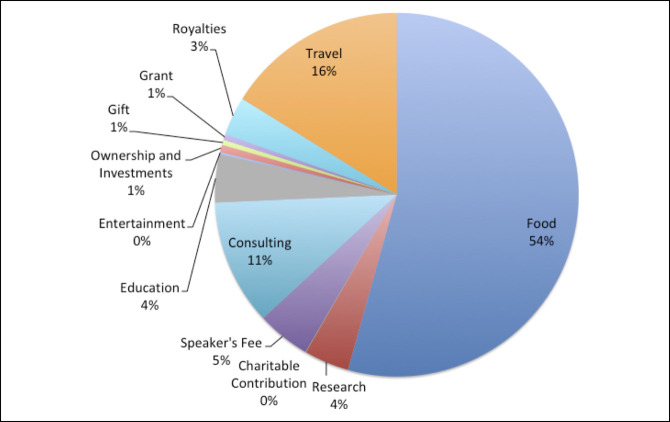
Illustration showing the distribution of Open Payments Database disclosures.

Initially, 2,503 AAOS presenters were identified, and 1,380 presenters remained for analysis after applying the exclusion criteria (Figure 1). Of the 1,380 presenters, 578 (41.9%) presented only a poster, 555 (40.2%) presented only a podium, and 247 (17.9%) presented both. Furthermore, of the authors included in this study, 682 (49.4%) were first authors while 698 (50.6%) were last authors. In addition, of those included, 440 presenters (31.9%) were from the Northeast, 326 (23.6%) were from the Midwest, 374 (27.1%) were from the South, and 240 (17.4%) were from the West. Author-affiliated specialties included adult reconstruction 336 (24.4%), foot and ankle 60 (4.4%), hand 81 (5.9%), tumor 57 (4.1%), pediatrics 91 (6.6%), practice management 102 (7.4%), shoulder and elbow 175 (12.7%), spine 142 (10.3%), sports 159 (11.5%), and trauma 177 (12.8%). Finally, of the authors included, 736 (53.3%) had at least one AAOS disclosure and 482 (34.9%) had at least one inconsistency. The distribution of types of disclosures in the AAOS disclosure program is shown in Figure 2 while the distribution of types of disclosures in the Open Payments Database is shown in Figure 3.

### Types of Inconsistencies

Inconsistencies between the AAOS disclosures and the Open Payments Database for each of the compared categories are summarized in Table 2. Using AAOS disclosures as the benchmark for comparison as previously described, 105 (7.6%) had an inconsistency within royalties, 133 (9.6%) had an inconsistency within speaker's bureau or paid presentations, 211 (15.3%) had an inconsistency within paid consultants, 215 (15.6%) had an inconsistency within research support, 192 (13.9%) had an inconsistency within stock or stock options, and 11 (0.8%) had an inconsistency within other financial or material support (Table 2). Overall, 482 (34.9%) had at least one inconsistency in each of these categories.

### Author Characteristics Associated with AAOS Disclosures or Inconsistencies

On univariate analysis, there was a significant difference in AAOS disclosure rates between author specialities (*P* = 0.002), author presentation type (*P* = 0.001), and author order (*P* < 0.001) (Table 3). Similarly, there was a significant difference in the inconsistency rate between author specialties (*P* = 0.006), author presentation type (*P* = 0.006), and author order (*P* < 0.001) (Table 3). In addition, the presence of an AAOS disclosure was also significantly associated with having an inconsistency (*P* < 0.001) (Table 3). Furthermore, authors with at least one inconsistency tended to have a higher number of poster presentations (*P* = 0.008), higher number of podium presentations (*P* = 0.025), higher total number of presentations (*P* < 0.001), higher number of AAOS disclosures (*P* < 0.001), and higher total value of payments in the Open Payments Database (in dollars) (*P* < 0.001) (Table 4).

**Table 3:** Inconsistencies Between the Open Payments Database and the AAOS Disclosure Database

Variable	AAOS disclosure rate, %	*P*-Value	Inconsistency rate, %	*P*-Value
Region of the United States		0.807		0.835
Northeast	55.0		35.0	
Midwest	53.4		33.4	
South	52.7		36.6	
West	51.3		34.2	
Specialty		**0.002**		**0.006**
Hand	40.7		22.2	** **
Foot and ankle	68.3		40.0	** **
Adult reconstruction	51.5		37.5	** **
Spine	62.7		47.9	** **
Shoulder and elbow	56.6		34.3	** **
Sports	53.5		34.6	** **
Trauma	47.5		30.5	** **
Tumor	45.6		26.3	** **
Pediatrics	63.7		36.3	** **
Practice management	47.1	** **	28.4	** **
Presentation type		**0.001**		**0.006**
Podium	51.4		33.2	** **
Poster	50.7		32.9	** **
Both	64.0	** **	43.7	** **
Author order		**<0.001**		**<0.001**
First	26.8		17.0	** **
Last	79.2		52.4	** **
Presence of an AAOS disclosure		N/A		**<0.001**
No			0.0	
Yes			65.5	

AAOS = American Academy of Orthopaedic Surgeons

Data in bold indicate statistical significance (*P* < 0.05).

**Table 4:** Comparison of Authors With and Without Inconsistencies Between the AAOS and Open Payments Databases

Variable	With Inconsistencies	Without Inconsistencies	*P*-Value
No. of poster presentations	Average: 1.0 ± 1.3	Average: 0.8 ± 0.9	**0.008**
No. of podium presentations	Average: 0.9 ± 1.2	Average: 0.8 ± 0.9	**0.025**
Total no. of presentations	Average: 1.9 ± 1.9	Average: 1.5 ± 1.3	**<0.001**
Total no. of AAOS disclosures	Average: 7.7 ± 6.4	Average: 0.8 ± 1.9	**<0.001**
Total value in open payments ($)	Average: $258,712 ± 657,651	Average: $47,979 ± 358,704	**<0.001**

AAOS = American Academy of Orthopaedic Surgeons

Data in bold indicate statistical significance (*P* < 0.05).

On stepwise multivariable Poisson regression, independent factors associated with having an AAOS disclosure included author specialty (*P* < 0.001), number of posters (*P* < 0.001), number of podiums (*P* = 0.005), and author order (*P* < 0.001) (Table 5). On stepwise multivariable Poisson regression, independent factors associated with having an inconsistency included number of AAOS disclosures (*P* < 0.001) and author order (*P* < 0.001) (Table 5).

**Table 5:** Factors Associated With AAOS Disclosure and Inconsistencies

Variable	Relative risk	95% CI	*P*-Value
With versus without AAOS disclosures			
Specialty			**<0.001**
Hand	Ref.		
Foot and ankle	1.59	1.23-2.06	
Adult reconstruction	1.31	1.05-1.63	
Spine	1.52	1.20-1.91	
Shoulder and elbow	1.46	1.15-1.84	
Sports	1.29	1.01-1.64	
Trauma	1.23	0.96-1.57	
Tumor	1.03	0.76-1.40	
Pediatrics	1.45	1.12-1.87	
Practice management	1.16	0.88-1.54	
No. of posters	1.06	1.03-1.10	**<0.001**
No. of podiums	1.05	1.01-1.08	**0.005**
Author order			** **
First		Ref.	
Last	2.91	2.56-3.30	**<0.001**
With versus without inconsistencies			
No. of AAOS disclosures	1.07	1.06-1.08	**<0.001**
Author order			** **
First		Ref.	
Last	1.90	1.60-2.26	**<0.001**

AAOS = American Academy of Orthopaedic Surgeons

Data in bold indicate statistical significance (*P* < 0.05).

### Total Payments by Specialty

The average total payment for hand was $22,497, for foot and ankle was $112,212, for adult reconstruction was $226,088, for spine was $154,015, for shoulder and elbow was $200,574, for sports was $115,703, for trauma was $60,494, for tumor was $52,716, for pediatrics was $30,033, and for practice management was $50,726. These differences in average total payments were statistically significant (*P* = 0.009). Of note, these are average total payments of the first and last authors for a presentation, as defined in the inclusion criteria.

## Discussion

The results of this study demonstrated that the overall inconsistency rate between physician-reported disclosures at a recent AAOS Annual Meeting and industry-reported relationships reported in the Open Payments Database has improved over the years to 35%. In an analysis of the AAOS 2014 Annual Meeting, Hannon et al. determined the discrepancy between disclosures at the AAOS 2014 Meeting and the Open Payments Database at that time to be 39%.^7^ Furthermore, this study also determined that the inconsistency rate within each disclosure category has improved as well. Although Hannon et al. noted an inconsistency rate of 11% within royalty relationships, 15% in speaker's fees, 24% in consultant fees, and 4% within other financial relationships, these rates have improved to inconsistencies of 8% in royalty relationships, 10% in speaker's fees, 15% in consultant fees, and 1% in other financial relationships in this study.^7^ In addition, this study was also able to identify an inconsistency rate of 16% for research support and 14% for stock or stock options. It should be emphasized that the previous study by Hannon et al. was unable to determine inconsistencies in the research support and stock or stock options categories.^7^ This was because at the time of their study, data in these categories in the Open Payments Database were incomplete and could not be analyzed. Therefore, inconsistency data in these categories have not been previously reported in the literature.

The Open Payments Database, which was first published in September 2014, was subject to several initial criticisms, including missing data and limited physician registration.^1^ Since then, there have been numerous improvements in the accuracy and reporting of data with more recent releases when compared with prior editions.^17^ Furthermore, specifically in the field of orthopaedic surgery, recent studies have demonstrated that both the number of surgeons receiving industry payment and the amount of individual payments received have markedly increased from 2014 to 2019.^18,19^ Thus, although Hannon et al. examined the differences in disclosures using data from the 2014 Open Payments Database, the present study offers a more accurate, contemporary analysis of disclosure inconsistencies. In addition, this study was also able to provide previously unreported data on the two aforementioned categories, research support and stock or stock options.

Several reasons could account for discrepancies in disclosures. First, there are different rules and guidelines governing disclosures to the AAOS Annual Meeting and the Open Payments Database. Some private companies are not required to disclose their payments through the Open Payments Database, and while the Open Payments Database discloses solely monetary relationships, the AAOS disclosure program discloses nonmonetary relationships as well.^7^ Finally, individual physicians often have differences in the understanding of disclosure requirements. For the AAOS Annual Meeting, the onus for the disclosure is on the physician, and thus, a surgeon may mistakenly omit or include relationships not provided in the Open Payments Database. A previous study by Okike et al.^20^ determined that the most common reasons for nondisclosure included payment unrelated to the topic of presentation at the meeting, a misunderstanding of the disclosure requirements, and accidental omission by the meeting program.

This study demonstrated that, on univariate analysis, certain factors, such as subspecialty type, type of presentation, number of poster or podium presentations, author order, number of AAOS disclosures, and total value of payments in the Open Payments Database, were associated with a higher rate of inconsistencies. Notably, spine surgeons had the highest rate of inconsistencies at 47.9%, which was similar to that reported by previous studies. In an analysis of the 2011 North American Spine Society Annual Meeting, Buerba et al. identified a 46% discrepancy between disclosures at the meeting and payments reported by the industry.^21^ Although certain orthopaedic subspecialties, most notably adult reconstruction and spine, have been shown to be a predictor of greater industry payments, it is unclear to why spine in particular has more inconsistencies than other orthopaedic subspecialties.^22-24^ On multivariable analysis, only two factors, number of AAOS disclosures and author order, particularly the last senior author position, were deemed to be independent risk factors for having an inconsistency. These two factors both suggest status as an influential figure in orthopaedic surgery. Thus, these individuals naturally have more relevant relationships with industry, which thus increases the likelihood of inconsistencies and errors in disclosures.

This study is not without limitations. There are differences in reporting structures of disclosures between the Open Payments Database and the AAOS Annual Meeting disclosure program. Notably, the AAOS Annual Meeting requires both monetary and nonmonetary industry relationships to be disclosed, whereas the Open Payments Database requires solely monetary disclosures.^7^ Furthermore, the AAOS Annual Meeting was chosen as the only comparison with the Open Payments Database because of its long-standing history in providing accurate disclosures of orthopaedic surgeons.^25^ There are alternative methods to track disclosures, such as high-impact journal publications and disclosures from orthopaedic subspecialty meetings, which could certainly yield different results than this study.^26,27^ In addition, AAOS disclosures were chosen as the benchmark to compare the Open Payments Database. However, the authors do recognize that neither the AAOS disclosures nor the Open Payments Database is a fully accurate representation of financial relationships for each physician. Furthermore, the Open Payments Database published payments from January 1, 2019, to December 31, 2019, but the 2019 AAOS Annual Meeting was held from March 12 to 16, 2019. However, because AAOS disclosures were chosen as the benchmark, effects of this timing discrepancy should be minimal. Furthermore, it is not possible to determine the magnitude in dollars of missing disclosures because the AAOS disclosures do not capture dollar amounts. The AAOS disclosures only have data on whether a disclosure was made.

To conclude, this study provides a thorough, contemporary analysis of inconsistencies in orthopaedic surgeon disclosures between the AAOS 2019 Annual Meeting and the Open Payments Database. The rate of inconsistencies between individual disclosure areas such as royalty payments, speaker fees, consultant fees, and other financial relationships were an improvement over previously reported data. However, despite this progress, it is still important for orthopaedic surgeons to review the Open Payments Database for its accuracy to provide the most transparent relationship possible between themselves and the industry.
